# Evaluation of Demographics and Social Life Events of Asian (*Elephas maximus*) and African Elephants (*Loxodonta africana*) in North American Zoos

**DOI:** 10.1371/journal.pone.0154750

**Published:** 2016-07-14

**Authors:** Natalia A. Prado-Oviedo, Mary K. Bonaparte-Saller, Elizabeth J. Malloy, Cheryl L. Meehan, Joy A. Mench, Kathy Carlstead, Janine L. Brown

**Affiliations:** 1 Center for Species Survival, Smithsonian Conservation Biology Institute, Smithsonian National Zoological Park, Front Royal, Virginia, United States of America; 2 Department of Environmental Science and Policy, George Mason University, Fairfax, Virginia, United States of America; 3 Department of Animal Science and Center for Animal Welfare, University of California Davis, Davis, California, United States of America; 4 Department of Mathematics and Statistics, American University, 4400 Massachusetts Ave. NW, Washington, District of Columbia, United States of America; 5 AWARE Institute, Portland, Oregon, United States of America; 6 Independent Researcher, Portland, Oregon, United States of America; University of Florida, UNITED STATES

## Abstract

This study quantified social life events hypothesized to affect the welfare of zoo African and Asian elephants, focusing on animals that were part of a large multi-disciplinary, multi-institutional elephant welfare study in North America. Age was calculated based on recorded birth dates and an age-based account of life event data for each elephant was compiled. These event histories included facility transfers, births and deaths of offspring, and births and deaths of non-offspring herd mates. Each event was evaluated as a total number of events per elephant, lifetime rate of event exposure, and age at first event exposure. These were then compared across three categories: species (African vs. Asian); sex (male vs. female); and origin (imported vs. captive-born). Mean age distributions differed (p<0.05) between the categories: African elephants were 6 years younger than Asian elephants, males were 12 years younger than females, and captive-born elephants were 20 years younger than imported elephants. Overall, the number of transfers ranged from 0 to 10, with a 33% higher age-adjusted transfer rate for imported African than imported Asian elephants, and 37% lower rate for imported females than males (p<0.05). Other differences (p<0.05) included a 96% higher rate of offspring births for captive-born females than those imported from range countries, a 159% higher rate of birthing event exposures for captive-born males than for their imported counterparts, and Asian elephant females being 4 years younger than African females when they produced their first calf. In summarizing demographic and social life events of elephants in North American zoos, we found both qualitative and quantitative differences in the early lives of imported versus captive-born elephants that could have long-term welfare implications.

## Introduction

Social events that occur throughout an animal’s lifetime, henceforth referred to as social life events, exert significant influences on its behavior, physiology, development, and overall welfare. For example, the birth of offspring can add to the dynamic nature of group interactions by increasing play [1] and the expression of nurturing behaviors [2, 3]. Disrupting stable social groups by adding or removing individuals via birth, death, or translocation can cause social instability and increased aggression [4, 5,6], resulting in elevated glucocorticoid levels [6, 7, 8] and subsequent immunosuppression [8, 9, 10] of group members. For offspring, premature separation from the mother, either through death or translocation, has been associated with increased short-term anxiety and stress [11], as well as longer-term effects such as poorer social skills [12, 13] and the development of abnormal (stereotypic) behaviors [14]. While the relationship between social life events and welfare have significant implications for the management of captive social species, the precise effects (direction, magnitude, and duration) of specific social life events will depend on the social complexities and natural history of each particular species as well as individual coping styles.

Both Asian and African elephants exhibit complex and elaborate patterns of sociality in the wild. At the center of their social systems are matrilineal core groups composed of genetically related adult females and their dependent offspring [15, 16, 17]. Females typically remain in these core groups while males disperse upon reaching adolescence. Although better documented in African elephants, dispersed males of both species remain primarily solitary or form fluid bachelor groups that separate when one or more males enter musth [18, 19, 20]. The adult females and offspring remaining in the core groups establish strong social relationships with one another, with benefits including increased protection against perceived threats, mutual care of calves, and aid to injured or fallen group members [21–26]. In general, African savannah elephants live in relatively large core groups consisting of 10–24 individuals [27, 28]. Population expansion over time results in fission of daughter groups that nevertheless continue to maintain close associations, leading to a complex hierarchical ‘fission-fusion’ social structure [15]. In contrast, Asian elephants typically live in smaller groups ranging from 3–7 individuals [17]. Additionally, Asian adult females have weaker ties with family groups and associate with maternal relatives only about 20% of the time [29, 30]. This suggests a different model for Asian elephants, in which family group fission leads to daughter groups that become largely independent of each other with inter-group transfers of females being infrequent [29, 30].

Studies of wild elephants indicate that elephant behavior and physiology can be strongly influenced by specific social life events, particularly those involving births or disruption of mother-offspring relationships. For example, when calves are born into a social group, non-maternal females may participate in allomothering, which can improve their abilities to successfully mother their own calves in the future [23]. The mother-offspring unit forms the basis of both African and Asian elephant society [16, 27], and calves are highly dependent on mothers for proper social development [31]. Premature mother-calf separations via poaching or culling have been associated with decreased social discrimination abilities and increased inter-species aggression among the orphans [32, 33, 34]. Loss of herd mates can have physiological and behavioral consequences for non-offspring as well. In east Africa, elephant females from disrupted social groups where poachers killed the matriarch or another integral core group member, displayed weaker social bonds and higher levels of fecal glucocorticoids compared to individuals of undisturbed groups [35]. Wild elephants also have been observed expressing directed empathetic behaviors when deceased conspecifics or herd mates are encountered [21, 22, 36], which suggests that death, in addition to disrupting social groups, can be an emotionally challenging experience for individual elephants, particularly if it results in the dissolution of strong bonds. These patterns suggest that social factors will also be important for the welfare of captive elephants. There is evidence that zoo elephants that spend more time in larger social groups, and particularly groups that include juveniles, have a reduced risk of performing stereotypic behavior [37]. Our goal was to provide data to further explore the relationships between social factors and the welfare of zoo elephants by quantifying social life events in the living North American zoo elephant population via use of studbooks.

Studbooks are record keeping tools that play a central role in organizing population data in a manner that supports informed management of *ex situ* populations [38]. Demographic studies have used studbook data to understand life history variables in order to improve captive breeding [39–44]. The information recorded in the American Zoo and Aquarium Association (AZA) Asian and African elephant studbooks allowed us to conduct the first-ever characterization of the living North American population in terms of species, sex, age distribution, and origin (i.e., imported from range countries or captive-born). We evaluated relationships between these population demographic characteristics and social life event variables (described in detail below) to discern patterns that could be relevant to elephant welfare as reported in the literature. For example, a study investigating disease transmission in the North American elephants found high levels of direct and indirect contacts within the population [45]. Suggesting that even for a species such as elephants, that have relatively low rates of inter-zoo transfers, the risk of disease transmission can be high [45]. With respect to species, investigation of the European zoo population demonstrate that age of separation and lifetime number of transfers are associated with a higher risk of mortality for Asian, but not African, females [46]. As such, the age at which social life events are first experienced by elephants, as well as the incidence rates of events based on demographics, could be important from a welfare standpoint.

Our analysis of information contained in the AZA Asian and African Elephant Studbooks aims to quantify variables that are important social life events for captive elephants. These variables are being used in further multivariable analyses of zoo elephant welfare that apply epidemiological methods to determine what factors in zoos are associated with several health and welfare outcomes [47], including rates of stereotypies [37], pituitary and ovarian endocrine function [48], and immunological markers and disease incidence. All of these are welfare indicators that can be influenced by life experiences [49–51].

## Methods

### Ethics Statement

This study was authorized by the management at each participating zoo and, where applicable, was reviewed and approved by zoo research committees. In addition, the study protocol was reviewed and approved by the Zoological Society of San Diego Institutional Animal Care and Use Committee N.I.H. Assurance A3675-01; Protocol 11–203. Our study was non-invasive.

### Subjects

All elephants in our study population met the following criteria: 1) housed in an AZA-accredited zoo in North America; 2) enrolled in the Using Science to Understand Zoo Elephant Welfare study [47]; 3) born prior to January 1, 2012; 4) alive as of December 31, 2012; 5) did not experience an inter-zoo transfer during 2012; and 6) had available studbook records. Our study population included 250 elephants from 68 zoos, which represents 83% of the total population of elephants housed in AZA-accredited North American zoos during 2012. Zoos that housed only Asian elephants made up 44% (30/68) of the participating institutions, 49% (33/68) of the participating institutions housed only African elephants, while only 7% (5/68) of the participating facilities housed mixed species (African and Asian) herds at the time of this study.

### Data Collection

We collected demographic data from the 2012 African and Asian Elephant North American Regional Studbooks, using information from between 2 March 1978 or 14 April 1962 (birth dates of the oldest African and Asian elephants in the study, respectively) and 31 Dec 2011.We imported the relevant data into Microsoft Excel (Microsoft Corporation, Redmond, WA). We defined each elephant according to three demographic categories: species (African, Asian); sex (male, female) and origin (imported, captive-born). Elephants were categorized as captive-born if they were recorded as having been born in a captive facility (in the U.S. or in a range country), or as imported if a capture location and date of capture was recorded in the studbook. We also calculated the age of each elephant as of 1 July 2012 from the known or estimated date of birth. Table 1 provides an overview of these population demographics.

**Table 1:** Summary of population demographics including number of elephants (N) and age distributions (mean, median, SEM, minimum and maximum) for each species by sex and origin.

				Age				
Species			N	Median	Mean	SEM	Minimum	Maximum
	** **	**Captive-born**	14	6.18	12.54	3.30	0.95	34.33
** **	**Female**	**Imported**	95	33.01	33.72	0.64	20.50	52.50
** **	** **	**All**	109	32.19	31.00	0.97	0.95	52.50
** **	** **	**Captive-born**	15	6.70	8.39	2.04	1.08	31.33
**African**	**Male**	**Imported**	10	29.50	29.29	1.71	21.50	37.94
** **	** **	**All**	25	11.91	16.75	2.50	1.08	37.94
** **	** **	**Captive-born**	29	6.53	10.39	1.92	0.95	34.33
** **	**Total**	**Imported**	105	32.50	33.29	0.61	20.50	52.50
** **	** **	**All**	134	30.60	28.34	1.03	0.95	52.50
** **	** **	**Captive-born**	28	21.84	21.17	2.33	1.02	48.77
** **	**Female**	**Imported**	63	42.50	43.69	0.98	19.50	64.50
** **	** **	**All**	91	40.50	36.76	1.47	1.02	64.50
** **	** **	**Captive-born**	16	13.87	18.84	3.86	2.16	50.21
**Asian**	**Male**	**Imported**	9	42.05	39.71	2.69	24.50	47.50
** **	** **	**All**	25	27.50	26.35	3.32	2.16	47.50
** **	** **	**Captive-born**	44	19.58	20.32	2.02	1.02	50.21
** **	**Total**	**Imported**	72	42.50	43.19	0.93	19.50	64.50
** **	** **	**All**	116	39.50	34.52	1.41	1.02	64.50

### Social Life Event Variables

We extracted social life event data (see definitions in Table 2) from the studbooks and organized them chronologically. We included all events that occurred after an elephant entered captivity (via importation or birth). Events that occurred prior to importation into the North American population are not represented in these data. Because we relied exclusively on information recorded in the studbooks, it is important to note two potential limitations in our datasets. First, there may have been gaps in the studbook records; thus, event data might under-represent some life events. In addition, because some elephants are managed as sub-groups and therefore not all elephants at a zoo necessarily interact physically with each other [52], we could not confirm that elephants were physically present for events involving births or deaths of herd-mates, except in cases of females who gave birth to offspring. Therefore, these data may over- represent individual exposures to births and deaths. To account for this, we differentiated between events that an elephant physically experienced (e.g., transfers and the birth of offspring for females) and those that they may have only been exposed to because of co-location (e.g., births and deaths of herd mates).

**Table 2:** Description of social life events recorded in studbooks.

Event Name	Abbreviation	Description
Age at Separation		The age of mother-offspring separations for captive-born elephants due to death of mother, transfer of mother or transfer of offspring
Transfers		Physical facility changes
Offspring Birth	OffB	Births of offspring to reproductively aged females (>8 yrs. old for African females; >5 yrs. old for Asian females)
Offspring Death	OffD	Deaths of offspring of parous females
Non-Offspring Birth Exposure (Females)	ExpB(f)	Non-offspring births at the same facility as female focal animals
Birth Exposure (Males)	ExpB(m)	Births (including offspring) at the same facility as male focal animals
Non-Offspring Death Exposure (Females)	ExpD(f)	Non-offspring deaths at the same facility as female focal animals
Non-Offspring Death Exposure (Males)	ExpD(m)	Deaths (including offspring) at the same facility as male focal animals

#### Variable Calculations

Age at Separation

We determined the date that captive born elephants (offspring) were separated from their mothers by comparing the chronological timelines of both. Separations were identified if there was either a facility transfer of the offspring (without the mother), a facility transfer of the mother (without the offspring) or the death of the mother. We calculated the age of the offspring at the time of separation based on the recorded date of the event and the birth date.

Transfers

We defined transfer events as recorded physical location changes excluding wild capture but including a transfer from the first facility in which an imported elephant was housed after capture (i.e., at importation). Transfers in ownership that did not involve a change in physical location were confirmed as such with the studbook managers, and were not counted. Variables based on transfer events included: 1) total count (number of transfers recorded between the date of captive birth or importation and December 2011); and 2) age at first transfer.

Offspring Births

Offspring birth events (OffB) were recorded births of offspring to reproductively aged females, defined as 8–52.5 years for Africans, and 5–64.5 years for Asians [48, 53]. We included all recorded offspring births regardless of how long the offspring lived. Variables based on OffB events included: 1) total count (number of OffB events recorded between the date the female reached reproductive age and December 2011); and 2) age at first offspring birth.

Offspring Deaths

Offspring death events (OffD) were recorded deaths of the offspring of parous females. Offspring deaths were counted if the offspring was housed at the same facility as the mother at the time of death, regardless of how long the offspring lived. Variables based on OffD events included: 1) total count (number of death events recorded between the date of the first recorded offspring birth and December 2011); and 2) age at first offspring death).

Birth Exposures

Birth exposures (ExpB) were recorded births of herd mates (elephants housed at the same zoo). These events were included regardless of how long the calf survived and differentiated between birth events that the elephant physically experienced (offspring births of a female) and herd mate birthing events to which that elephant may or may not have been exposed. Therefore, for males ExpB events include the birth of their own offspring as well as births of herd mates, whereas for females this variable only includes herd mate births. Variables based on these events included: 1) total count (the number of these events recorded between the date of birth or importation and December 2011); and 2) age at first ExpB event.

Death Exposures.

Death exposures (ExpD) are recorded deaths of herd mates (elephants housed at the same zoo). All deaths were counted regardless of age at death. For males, ExpD events included the deaths of their own offspring as well as deaths of herd mates, whereas for females ExpD only included herd mate deaths. Variables based on ExpD events included: 1) total count (the number of events recorded between date of birth or importation through December 2011); and 2) age at first ExpD event.

### Statistical Analysis

The normal distribution assumption was not met by the population age structure or by any of the life event variables. The variables (total counts and age at first recorded event) were strongly positively skewed for each event type due to the presence of “0” total events (i.e., an event never occurred in the lifetime of the elephant). Zero events were included in the analyses where appropriate because in certain cases we considered a lack of experience/exposure to be biologically relevant (see discussion). Mann-Whitney U tests were performed to determine rank order differences in the age (as of 7/1/2012) of elephants across the different demographic categories, and differences in the age at separation across species and sex.

The reference categories were Asian, male and imported for all the regression models we tested. Significant main effects and two-way interactions are reported from regression models. We show the interaction terms at the factor level (African vs. Asian, female vs. male, imported vs. captive-born) to demonstrate how they interact by looking at the specific effects for each combination. Multi-variable regression models were used to determine associations between variable event rates and the demographic categories. For the transfer, offspring birth and death event variables, Poisson regression models were used because the variance approximately equaled the mean. The birth and exposure events showed evidence of over-dispersion, with a deviance twice the degrees of freedom. Therefore, negative binomial models were used to analyze these events. Poisson and negative binomial models incorporated the total counts of all the event variables, using the natural log of age (in years) as the time/exposure variable and all three demographic categories were included as factors. We tested models for multi-collinearity using Variance Inflation Factor scores with a cutoff value of 2. All independent variables were included in initial models as main effects. Where significant main effects were found, two and three-way interactions were also tested. In models with offspring deaths (females only) and herd mate deaths (both male and female), birth events were included as covariates in order to take into account how these events impact the rate of death exposures. Factors that did not improve the overall predictive power of the models were dropped sequentially until only those that significantly estimated rates remained. Three-way interactions between species-sex-origin were tested, but none were significant and they are therefore not reported. The β coefficient estimate is the natural logarithm of the incident rate ratio (IRR) of exposure to the event.

A Kaplan–Meier analysis was performed to determine differences in the recorded age at first event for each event type across the demographic categories. Age at first event was calculated from the known or estimated date of birth to the recorded date of the event occurrence. Kaplan–Meier analysis can take into account right-censored data. However, due to the large proportion of reproductively aged females that have never given birth, including all the females in the offspring birth event variable would not accurately reflect the biological age at which female elephants gave birth in the population. Therefore, zero events were excluded for the offspring birth life event only. Due to the exclusion of reproductively aged females that had not yet experienced birthing events at the time of the study, only a subset of elephants was used in the offspring birth analysis. This subset therefore differed in the number of elephants included in the subsequent OffB Cox regression model for the OffB variable (see below).

Multi-variable Cox regression models were used to determine the associations between demographic category and the hazard ratio (or relative risk) of the first social life event recorded in the studbook. Cox regression models incorporated the first occurrence of each event, using the natural log of age at first event (in years) as the time/exposure variable; all three demographic categories were included as factors. Two- and three-way interactions between the independent variables were tested, but were not significant and are therefore not reported. The full models of the main effects are reported, where β is the estimated regression coefficient for the model. The β coefficient estimate is the natural logarithm of the estimated relative risk (ERR) of exposure to the first event. A positive coefficient indicates a higher estimated relative risk and a negative coefficient indicates a lower risk for the demographic category with which the event is associated. Data were right-censored due to the inclusion of individuals that had not yet experienced certain life events by the end of the study period, meaning that individuals with zero events in their histories were included in the Cox regressions and therefore influenced the risk predicted in the models. Statistical analyses were conducted using SPSS [54]; p<0.05 was considered statistically significant.

## Results

Table 3 shows the social life event data and age distribution for the population as a whole and broken down by species, sex and origin. Asian elephants were older (p<0.001) than African elephants, female elephants were older (p<0.001) than male elephants, and captive-born elephants were younger (p<0.001) than imported elephants.

**Table 3:** Summary of life events and age (as of 7/1/2012) for the full population (n = 250) by species, sex and origin. Data include the number of elephants that were included in each variable (n), and the number of events, including median, mean (# events/elephant), SEM, minimum and maximum.

** **	**Full Population**													
	**n**	**Total Counts**	**Median**	**Mean**	**SEM**	**Min**	**Max**							
**Age**	250	-	33.02	31.21	0.88	0.95	64.50							
**Transfers**	250	668	2.00	2.67	0.13	0	10.00							
**OffB**	188	104	0.00	0.54	0.08	0	6.00							
**OffD**	54	48	1.00	0.89	0.15	0	5.00							
**ExpB(m)**	50	206	3.00	4.12	0.73	0	26.00							
**ExpD(m)**	50	167	2.00	2.34	0.60	0	19.00							
**ExpB(f)**	200	532	1.00	2.66	0.30	0	21.00							
**ExpD(f)**	200	727	2.00	3.63	0.35	0	29.00							
** **	**Species**													
	**African Elephants**							**Asian Elephants**						
	**n**	**Total Counts**	**Median**	**Mean**	**SEM**	**Min**	**Max**	**n**	**Total Counts**	**Median**	**Mean**	**SEM**	**Min**	**Max**
**Age**^**a**^	134	-	30.60	28.34	1.03	0.95	52.50	116	-	39.50	34.52	1.41	1.02	64.50
**Transfers**	134	364	2.00	2.72	0.17	0	10.00	116	304	2.50	2.62	0.19	0	10.00
**OffB**	101	40	0.00	0.38	0.07	0	3.00	87	64	0.00	0.74	0.14	0	6.00
**OffD**	26	15	0.00	0.58	0.15	0	3.00	28	33	1.00	1.18	0.24	0	5.00
**ExpB(m)**	25	76	2.00	3.04	0.60	0	10.00	25	130	3.00	5.20	1.31	0	26.00
**ExpD(m)**	25	51	1.00	2.04	0.40	0	7.00	25	116	3.00	4.64	1.08	0	19.00
**ExpB(f)**	109	189	1.00	1.73	0.24	0	10.00	91	343	1.00	3.77	0.58	0	21.00
**ExpD(f)**	109	264	2.00	2.42	0.25	0	15.00	91	463	3.00	5.09	0.69	0	29.00
** **	**Sex**													
	**Female Elephants**							**Male Elephants**						
	**n**	**Total Counts**	**Median**	**Mean**	**SEM**	**Min**	**Max**	**n**	**Total Counts**	**Median**	**Mean**	**SEM**	**Min**	**Max**
**Age**^**a**^	200	-	34.50	33.62	0.87	0.95	64.50	50	-	22.00	21.55	2.17	1.08	50.21
**Transfers**	200	561	3.00	2.80	0.13	0	10.00	50	107	1.00	2.14	0.36	0	10.00
**ExpB**	200	532	1.00	2.66	0.30	0	21.00	50	206	3.00	4.12	0.73	0	26.00
**ExpD**	200	727	2.00	3.63	0.35	0	29.00	50	167	2.00	3.34	0.60	0	19.00
** **	**Origin**													
	**Imported Elephants**							**Captive-Born Elephants**						
	**n**	**Total Counts**	**Median**	**Mean**	**SEM**	**Min**	**Max**	**n**	**Total Counts**	**Median**	**Mean**	**SEM**	**Min**	**Max**
**Age**^**a**^	177	-	36.58	37.32	0.63	19.5	64.50	73	-	13.59	16.38	1.54	0.95	50.21
**Transfers**	177	591	3.00	3.34	0.13	0	10.00	73	77	0.00	1.05	0.17	0	5.00
**OffB**	158	82	0.00	0.52	0.09	0	6.00	30	22	0.00	0.67	0.19	0	3.00
**OffD**	43	35	0.00	0.81	0.17	0	5.00	11	13	0.00	1.18	0.26	0	2.00
**ExpB(m)**	19	92	3.00	4.84	1.27	0	16.00	31	114	3.00	3.68	0.89	0	26.00
**ExpD(m)**	19	92	4.00	4.84	1.10	0	19.00	31	75	1.00	2.42	0.65	0	16.00
**ExpB(f)**	158	417	1.00	2.64	0.36	0	21.00	42	115	2.00	2.74	0.54	0	17.00
**ExpD(f)**	158	617	2.00	3.91	0.43	0	29.00	42	110	2.00	2.62	0.44	0	12.00

^a^Values differ within demographic category (p<0.05) (Mann-Whitney U Test). OffB = offspring births; OffD = offspring deaths; ExpB = exposure to births; ExpD = exposure to deaths; m = male; f = female.

### Age at Separation

Of the 73 captive-born elephants in the study, 65 had mothers that could be identified by studbook numbers. Of these, 38 individuals (58%, 38/65) were still at the facility with their mother at the time of the study. Of the 42% (27/65) no longer with their mothers, 18 were separated due to transfer of the individual, one due to transfer of the mother, and eight due to the death of the mother. The average age at separation for the full population was 9.32 years; data by sex and species are shown in Table 4. There were no species (p = 0.17) or sex (p = 0.94) differences.

**Table 4:** Age at separation (median, mean, SEM, minimum and maximum) from mother due to either transfer of individual, transfer of mother, or death of mother for the full population and by species and sex.

	**Full Population**											
	**n**	**Median**	**Mean Age**	**SEM**	**Min**	**Max**						
**Transfer of Individual**	18	5.30	6.60	1.30	1.30	25.00						
**Transfer of Mother**	1		11.20									
**Death of Mother**	8	14.10	15.20	4.70	1.50	35.00						
**All Separations**	27	6.10	9.30	1.80	1.30	35.00						
	**Species**											
	**African**						**Asian**					
	**n**	**Median**	**Mean Age**	**SEM**	**Min**	**Max**	**n**	**Median**	**Mean Age**	**SEM**	**Min**	**Max**
**Transfer of Individual**	5	10.40	11.60	3.70	2.20	25.00	13	4.20	4.60	0.80	1.30	11.10
**Transfer of Mother**	0						1		11.20			
**Death of Mother**	5	7.60	12.30	5.10	1.50	27.90	3	23.60	20.00	9.90	1.50	35.00
**All Separations**	10	12.00	10.10	3.00	1.50	27.90	17	5.00	7.80	2.20	1.30	35.00
	**Sex**											
	**Male**						**Female**					
	**n**	**Median**	**Mean Age**	**SEM**	**Min**	**Max**	**n**	**Median**	**Mean Age**	**SEM**	**Min**	**Max**
**Transfer of Individual**	10	6.60	7.90	2.20	1.30	25.00	8	4.20	5.00	1.10	2.20	11.10
**Transfer of Mother**	1		11.20				0					
**Death of Mother**	2	18.30	18.30	16.80	1.50	35.00	6	14.07	14.20	4.20	1.50	27.90
**All Separations**	13	7.60	9.70	2.80	1.30	35.00	14	5.30	8.90	2.30	1.50	27.90

### Transfers

**Fig 1:**
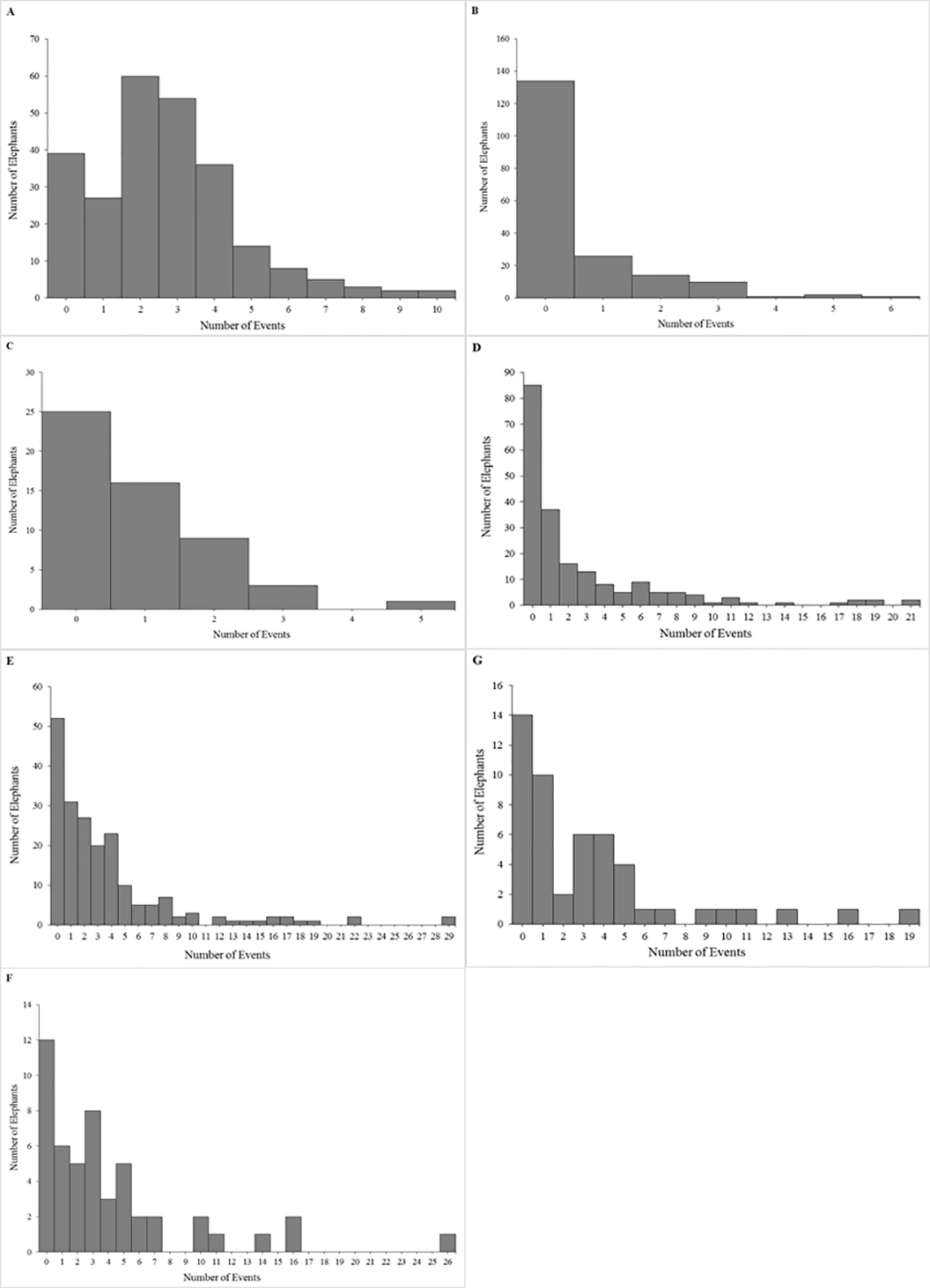
Summary of event frequencies for the study population of African and Asian elephants in North American zoos. Frequency of number of elephants experiencing (A) Transfers, (B) Offspring births–females, (C) Offspring Deaths–females, (D) Birth exposures–females, (E) Death exposures–females, (F) Death exposures–males, and (G) Birth exposures–males. Bins include no experience/exposure (0 events).

Of the total population, 84% (211/250) of elephants experienced at least one transfer event (Fig 1A). When we compared within the origin category, we found that imported African elephants had a 33% higher incident rate ratio for transfer events than imported Asian elephants (Tables 5 and 6). There was no species difference in the average age or in the estimated relative risk at which the first transfer event was experienced (Tables 7 and 8).

**Table 5:** Summary of parameter estimates from Poisson regression models, including beta estimates, standard errors (SE) and corresponding 95% confidence intervals. p<0.05.

		**Transfers**					
					**95% C.I.**		
		**N**	**β**	**IRR**	**Lower**	**Upper**	**P-Value**
**Imported—Species**	**African**	105	0.29	1.33	1.30	1.57	0.001
	**Asian**	72	Reference				
**Imported—Sex**	**Female**	158	-0.46	0.63	0.50	0.80	<0.001
	**Male**	19	Reference				
**Captive-born—Species**	**African**	29	-0.58	0.78	0.96	3.33	0.06
	**Asian**	44	Reference				
**Captive-born—Sex**	**Female**	42	0.30	0.26	0.45	1.22	0.24
** **	**Male**	31	Reference				
		**Offspring Births**					
					**95% C.I.**		
		**N**	**β**	**IRR**	**Lower**	**Upper**	**P-Value**
**Species**	**African**	101	-0.37	0.70	0.47	1.05	0.09
	**Asian**	87	Reference				
**Origin**	**Captive-born**	30	0.67	1.96	1.2	3.18	0.007
** **	**Imported**	158	Reference				
		**Offspring Deaths**					
					**95% C.I.**		
		**N**	**β**	**IRR**	**Lower**	**Upper**	**P-Value**
**Species**	**African**	26	0.31	1.36	0.61	3.04	0.46
	**Asian**	28	Reference				
**Origin**	**Captive-born**	11	1.00	2.72	1.28	5.77	0.009
	**Imported**	43	Reference				
**Covariate**	**OffB**	54	0.51	1.67	1.341	2.08	<0.001

**Table 6:** Summary of incident rate ratios (IRR) relative to time using Poisson regression models, including 95% confidence intervals for IRR. p<0.05. IRR = estimated rate of event experience/exposure.

		**Birth Exposures—Males**					
					**95% C.I.**		
		**N**	**β**	**IRR**	**Lower**	**Upper**	**P-Value**
**Species**	**African**	25	0.44	1.55	0.77	3.12	0.23
	**Asian**	25	Reference				
**Origin**	**Captive-born**	31	0.95	2.59	1.34	5.02	0.01
** **	**Imported**	19	Reference				
		**Death Exposures—Males**					
					**95% C.I.**		
		**N**	**β**	**IRR**	**Lower**	**Upper**	**P-Value**
**Species**	**African**	25	0.14	1.15	0.55	2.40	0.71
	**Asian**	25	Reference				
**Origin**	**Captive-born**	31	0.30	1.35	0.67	2.75	0.40
	**Imported**	19	Reference				
**Covariate**	**ExpB**	50	0.08	1.08	1.01	1.16	0.03
		**Birth Exposures—Females**					
					**95% C.I.**		
		**N**	**β**	**IRR**	**Lower**	**Upper**	**P-Value**
**Imported—Species**	**African**	95	-0.66	0.52	3.53	0.75	0.001
	**Asian**	63	Reference				
**Captive-Born—Species**	**African**	14	-1.01	0.36	0.17	0.80	0.01
	**Asian**	28	Reference				
**Asian—Origin**	**Captive-born**	28	0.46	1.59	0.93	2.70	0.09
	**Imported**	63	Reference				
**African—Origin**	**Captive-born**	14	1.67	5.34	2.22	12.83	<0.001
** **	**Imported**	95	Reference				
		**Death Exposures—Females**					
					**95% C.I.**		
		**N**	**β**	**IRR**	**Lower**	**Upper**	**P-Value**
**Species**	**African**	109	0.09	1.10	0.77	1.56	0.60
	**Asian**	91	Reference				
**Origin**	**Captive-born**	42	0.70	2.01	1.30	3.10	0.002
	**Imported**	158	Reference				
**Covariate**	**ExpB**	200	0.16	1.17	1.12	1.22	<0.001

**Table 7:** Summary of age (in years) at first recorded event exposure using Kaplan-Meier analysis including, number of elephants (N) included in the analyses, mean, SEM and median.

	**Full Population**											
	**N**	**Median Age**	**Mean Age**	**SEM**	**Min**	**Max**						
**Transfers**	211	2.51	6.77	0.84	0	45.92						
**OffB**	54	18.72	19.02	0.78	6.51	32.72						
**OffD**	31	20.94	21.74	1.35	6.51	39.52						
**ExpB(m)**	38	1.94	14.5	2.09	0.08	27.32						
**ExpD(m)**	36	6.10	7.85	1.10	0.05	22.87						
**ExpB(f)**	115	26.65	32.86	1.71	0	49.61						
**ExpD(f)**	148	11.58	12.86	0.82	0	49.77						
	**Species**											
	**African Elephants**						**Asian Elephants**					
	**N**	**Median Age**	**Mean Age**	**SEM**	**Min**	**Max**	**N**	**Median Age**	**Mean Age**	**SEM**	**Min**	**Max**
**Transfers**	113	2.42	4.66	0.66	0.25	24.96	98	3.08	8.33	1.36	0	45.92
**OffB**	26	21.72	21.3	1.13	10.42	32.72	28	15.69	16.9	0.94	6.51	25.82
**OffD**^**a**^	12	24.24	24.18	1.87	12.00	32.72	19	19.32	20.20	1.83	6.51	39.52
**ExpB(m)**	18	10.94	15.38	2.89	0.08	27.32	20	10.66	12.59	2.61	0.42	23.26
**ExpD(m)**^**a**^	18	5.03	8.56	1.81	0.72	22.87	18	6.60	7.14	1.30	0.05	20.65
**ExpB(f)**	60	27.71	29.75	1.84	0.19	49.61	55	24.46	32.89	2.44	0	45.95
**ExpD(f)**^**a**^	80	15.66	15.07	1.11	0.30	44.51	68	6.83	10.26	1.14	0	49.77
** **	**Sex**											
	**Female Elephants**						**Male Elephants**					
	**N**	**Median Age**	**Mean Age**	**SEM**	**Min**	**Max**	**N**	**Median Age**	**Mean Age**	**SEM**	**Min**	**Max**
**Transfers**^**a**^	178	2.26	5.73	0.81	0	45.92	33	5.00	11.36	2.69	0.03	37.31
**ExpB**	115	26.65	32.86	1.71	0	49.61	38	10.94	14.45	2.09	0.08	27.32
**ExpD**	148	11.58	12.86	0.82	0	49.77	36	6.10	7.85	1.10	0.05	22.87
	**Origin**											
	**Imported Elephants**						**Captive-Born Elephants**					
	**N**	**Median Age**	**Mean Age**	**SEM**	**Min**	**Max**	**N**	**Median Age**	**Mean Age**	**SEM**	**Min**	**Max**
**Transfers**^**a**^	177	2.00	3.25	0.40	0	45.92	34	10.41	18.8	3.31	0.40	24.96
**OffB**^**a**^	43	19.55	19.81	0.84	10.42	32.72	11	14.34	15.93	1.74	6.51	25.82
**OffD**^**a**^	22	22.64	23.28	1.54	12.00	39.52	9	17.45	17.98	2.46	6.51	28.43
**ExpB(m)**^**a**^	13	19.06	25.22	2.74	10.94	25.26	25	2.55	6.63	1.71	0.08	27.32
**ExpD(m)**^**a**^	16	12.51	12.68	1.61	1.71	22.87	20	2.75	3.99	0.80	0.05	14.50
**ExpB(f)**^**a**^	86	32.14	36.84	1.75	4.44	49.61	29	3.37	15.71	3.25	0	22.25
**ExpD(f)**^**a**^	118	14.63	14.77	0.9	0.30	49.77	30	2.64	5.32	1.23	0	27.14

^a^Values differ within demographic category (Kaplan-Meier Analysis) (p< 0.05). OffB = offspring births; OffD = offspring deaths; ExpB = exposure to births; ExpD = exposure to deaths; m = male; f = female.

**Table 8:** Summary of parameter estimates for estimated relative risk (ERR) of exposure to/experiencing the first recorded event using multi-variable Cox regressions, including 95% Wald confidence intervals for ERR. p<0.05. ERR = estimated relative risk of exposure to/experiencing the first event.

		**Transfers**											
					**95% C.I.**								
		**N**	**β**	**ERR**	**Lower**	**Upper**	**P-Value**						
**Species**	**African**	134	0.21	1.24	0.93	1.66	0.15						
	**Asian**	116	Reference										
**Sex**	**Female**	200	0.28	1.32	0.90	1.95	0.16						
	**Male**	50	Reference										
**Origin**	**Captive-born**	73	-1.50	0.22	0.15	0.33	<0.001						
** **	**Imported**	177	Reference										
		**Offspring Births**						**Offspring Deaths**					
					**95% C.I.**		** **				**95% C.I.**		
		**N**	**β**	**ERR**	**Lower**	**Upper**	**P-Value**	**N**	**β**	**ERR**	**Lower**	**Upper**	**P-Value**
**Species**	**African**	104	-0.24	0.79	0.45	1.38	0.41	26	-0.28	0.75	0.34	1.65	0.48
	**Asian**	87	Reference					28	Reference				
**Origin**	**Captive-born**	33	0.72	2.06	1.03	4.11	0.04	11	1.39	4.01	1.61	9.95	0.003
** **	**Imported**	158	Reference					43	Reference				
		**Birth Exposures—Males**						**Death Exposures—Males**					
					**95% C.I.**		** **				**95% C.I.**		
		**N**	**β**	**ERR**	**Lower**	**Upper**	**P-Value**	**N**	**β**	**ERR**	**Lower**	**Upper**	**P-Value**
**Species**	**African**	25	-0.45	0.64	0.33	1.26	0.19	25	0.06	1.07	0.55	2.07	0.85
	**Asian**	25	Reference					25	Reference				
**Origin**	**Captive-born**	31	1.76	5.84	2.70	12.61	<0.001	31	0.93	2.53	1.17	5.48	0.02
** **	**Imported**	19	Reference					19	Reference				
		**Birth Exposures—Females**						**Death Exposures—Females**					
					**95% C.I.**		** **				**95% C.I.**		
		**N**	**β**	**ERR**	**Lower**	**Upper**	**P-Value**	**N**	**β**	**ERR**	**Lower**	**Upper**	**P-Value**
**Species**	**African**	109	0.21	1.23	0.83	1.82	0.31	109	0.01	1.01	0.71	1.44	0.97
	**Asian**	91	Reference					91	Reference				
**Origin**	**Captive-born**	42	1.38	3.96	2.51	6.24	<0.001	42	0.89	2.44	1.57	3.8	<0.001
	**Imported**	200	Reference					200	Reference				

In the Poisson models when we compared within origin, imported females had a 37% lower transfer rate than imported males (p<0.001) (Tables 5 and 6). The Kaplan-Meier analyses demonstrated that males were on average older than females at the time of the first recorded transfer event (p<0.001) (Table 7), but the Cox model showed that there was no sex difference in the estimated relative risk of experiencing this first event. Because importation was counted as a transfer event, all imported elephants experienced at least one transfer event in their lifetime. In addition, 94% experienced at least one subsequent transfer post-importation. In contrast, 45% (33/73) of captive-born individuals had not experienced a transfer event (Table 2). Finally, captive-born elephants were older when they experienced their first transfer event (Table 7) and had a 78% lower estimated relative risk of experiencing this first event than imported elephants (Table 8).

### Offspring Births (OffB)

Only females of reproductive age (n = 188) were included in these analyses. Of this portion of the population, 29% (54/188) experienced at least one recorded offspring birth event (Fig 1B). There were no species differences in the age at which the first recorded event occurred (Table 7), or the estimated relative risk of experiencing a first birth (Table 8). Compared to imported females, captive-born females had an estimated 96% higher OffB incident rate ratio (Tables 5 and 6). Captive-born females were also younger than imported females when they experienced their first birth event (p<0.05) (Table 7), and had a 106% higher estimated relative risk for first offspring birth (Table 8).

### Offspring Deaths (OffD)

Only parous females (n = 54) were included in these analyses. Of this population, 54% (29/54) experienced at least one recorded offspring death event (Fig 1C). We accounted for the number of offspring births the females experienced and determined that for every offspring that was born there was a corresponding 67% increase in the rate of offspring deaths they experienced (p<0.001). There was no species difference in the incident rate ratio of these events. Although African elephants were older than Asian elephants at the time of their first event (p<0.05) (Table 9), there were no species differences in the estimated relative risk of experiencing offspring death (Table 10). When compared within the origin group, captive-born females had a 172% higher incident rate ratio than imported females (p = 0.009). Captive-born females were also younger when they experienced their first calf death (p<0.001) (Table 9), and had a 301% higher estimated relative risk for this event than imported females (Table 10).

**Table 9:** Summary of parameter estimates from negative binomial regression models, including beta estimates, standard errors (SE) and corresponding 95% confidence intervals. p<0.05.

		**Birth Exposures—Males**				
				**95% C.I.**		
		**β**	**SE**	**Lower**	**Upper**	**P-Value**
**Origin**	**Captive-born**	0.90	0.34	0.24	1.56	0.01
	**Imported**	Reference				
		**Death Exposures—Males**				
				**95% C.I.**		
		**β**	**SE**	**Lower**	**Upper**	**P-Value**
**Covariate**	**ExpB**	0.08	0.04	0.01	0.15	0.03
		**Birth Exposures—Females**				
				**95% C.I.**		
**Main Effects**		**β**	**SE**	**Lower**	**Upper**	**P-Value**
**Species**	**African**	-0.35	0.17	-0.69	-0.02	0.04
	**Asian**	Reference				
**Origin**	**Captive-born**	1.15	0.22	0.71	1.58	<0.001
	**Imported**	Reference				
**Two-Way Interactions**						
**Imported—Species**	**African**	-0.66	0.19	-1.04	-0.29	0.001
	**Asian**	Reference				
**Captive-Born—Species**	**African**	-1.01	0.40	-1.80	-0.22	0.01
	**Asian**	Reference				
**African—Origin**	**Captive-born**	1.67	0.45	0.80	2.55	<0.001
	**Imported**	Reference				
		**Death Exposures—Females**				
				**95% C.I.**		
		**β**	**SE**	**Lower**	**Upper**	**P-Value**
**Origin**	**Captive-born**	0.70	0.22	0.26	1.13	0.002
	**Imported**	Reference				
**Covariate**	**ExpB**	0.16	0.02	0.11	0.20	<0.001

**Table 10:** Summary of incident rate ratios (IRR) relative to time using negative binomial models, including 95% confidence intervals for IRR. p<0.05. IRR = estimated rate of event experience/exposure

		**Birth Exposures—Males**			
			**95% C.I.**		
		**IRR**	**Lower**	**Upper**	**P-Value**
**Origin**	**Captive-born**	2.59	1.34	5.02	0.01
	**Imported**				
		**Death Exposures—Males**			
			**95% C.I.**		
		**IRR**	**Lower**	**Upper**	**P-Value**
**Covariate**	**ExpB**	1.08	1.01	1.16	0.03
		**Birth Exposures—Females**			
			**95% C.I.**		
		**IRR**	**Lower**	**Upper**	**P-Value**
**Imported—Species**	**African**	0.52	3.53	0.75	0.001
	**Asian**				
**Captive-Born—Species**	**African**	0.36	0.17	0.80	0.01
	**Asian**				
**African—Origin**	**Captive-born**	5.34	2.22	12.83	<0.001
	**Imported**				
		**Death Exposures—Females**			
			**95% C.I.**		
		**IRR**	**Lower**	**Upper**	**P-Value**
**Origin**	**Captive-born**	2.01	1.30	3.10	0.002
	**Imported**				
**Covariate**	**ExpB**	1.17	1.12	1.22	<0.001

### Birth Exposures (ExpB)

Of the total population, 58% (115/200) female elephants experienced at least one recorded herd mate birth event (Fig 1D). When compared within the origin category, imported and captive-born African elephant females had a 48% (p = 0.001) and 64% (p = 0.01) lower incident rate ratio than their Asian elephant counterparts, respectively (Tables 9 and 10). For African elephants only, captive-born females had a 434% higher incident rate ratio for being exposed to a birth than imported females (p<0.001) (Tables 9 and 10). Compared to their imported counterparts, captive-born females were younger at the time of their first recorded exposure to a birth (p<0.001) (Table 7) and had a 296% higher estimated relative risk of being exposed to such an event (Table 8).

Total count of herd mate births was at least one for 76% (38/50) of the males (Fig 1G). Captive-born male elephants had a 159% higher incident rate ratio than imported males (Tables 6 and 9). While they did not exhibit any differences in their age at first event (p = 0.36) (Table 7), captive-born males had a 484% higher estimated relative risk than imported males of experiencing a first birthing event (Table 8).

### Death Exposures (ExpD)

Of the total population, 74% (148/200) of female elephants experienced at least one recorded herd mate death (Fig 1E). We accounted for the number of herd mate births females were exposed to and found that for every herd mate birth there was a corresponding 17% increase in the rate of herd mate deaths (Tables 9 and 10). African females were older than Asian female elephants the first time they experienced a death in the herd (p = 0.02) (Table 7). Captive-born female elephants had a 101% higher incident rate ratio of herd mate death events (p = 0.002) (Tables 9 and 10) than imported females, and were also older (p<0.001) at the time of the first event and had an estimated 144% higher estimated relative risk of experiencing this first event (Tables 7 and 8).

Of the total population 72% (36/50) of male elephants experienced at least one recorded herd mate death (Fig 1F). We accounted for the number of birth exposures and determined that for every herd mate birth male elephants were exposed to there was a corresponding 8% increase in the rate of herd mate deaths to which they were exposed (Tables 9 and 10). African male elephants were older than Asian male elephants when they experienced their first death event (p = 0.02) (Table 7), but they did not differ in their estimated relative risk of experiencing it (Table 8). Imported males were older than captive-born males at the time a first recorded death event occurred (p<0.001) (Table 7), and had an estimated 153% higher estimated relative risk of experiencing this first event (Table 8).

## Discussion

Our studbook analysis provides new information about the demographics of a substantial proportion of the living North American zoo elephant population, and sheds light on similarities and differences in social life histories of elephants with different demographic characteristics. In the discussion that follows, we interpret the life event analysis results both in the context of zoo management practices and with respect to their relevance to elephant welfare [37, 47, 48, 52, 55–58]. Some of the results, particularly when presented by sex and origin, should be interpreted with caution because of small animal numbers. Nevertheless, this study represents the most comprehensive analysis of social life events for elephants living in U.S. zoos.

### Population Demographics

Historically, zoos supplemented captive herds by importing wild calves from elephant orphanages (primarily Asians) or as a result of culling programs (Africans) in the 1950s and 60s [59]. According to the studbook, imported elephants make up 62% of the total Asian elephant population, with the most recent importation of an Asian elephant in 1996. Thus, addition of young elephants to the Asian elephant population for the past 20+ years has been through captive breeding [59–61]. By contrast, wild-born African elephants have been imported to the U.S. more regularly, most recently in 2003, and make up 78% of the total African elephant population. Overall, importations were heavily skewed towards females for both species, initially because they were considered easier to manage and display and more recently for breeding purposes. As a result, more than three quarters (79%, 158/200) of females in the study population were imported, compared to only 34% (17/50) of male elephants. Imported elephants also were on average 20 years older than captive-borns, reflecting the long generation interval of elephants and the fact that breeding in zoos was not widespread until the early 1990’s [59–61]. Thus, it was not surprising that males in general were younger than females. Nearly all African males (14/15) were under the age of 13 and most Asian males (10/16) were under the age of 20, while 71% (141/200) of female elephants were 30 or older. Taken together, the decrease in frequency of importation as well as low rates of captive births have led to a female population with an age structure that is heavily skewed toward older animals [62, 63, 64]. Older animals are more likely to experience health issues such as foot problems [56] and female reproductive problems, including ovarian acyclicity and hyperprolactinemia [48]. This emphasizes the importance of research to better understand the impact of aging on the health, welfare and reproduction of zoo elephants [65].

### Social Life Events, Age at Separation and Transfers

Prior to our study, data on transfers and age at separation had been collected for the European female zoo elephant population and used in analyses of survivorship and fecundity [66, 67]. Those studies highlighted the importance of evaluating life history in conjunction with demographics; for example, transfers reduced survivorship, but only in Asian females [66]. Our analysis differed in that it focused on the living North American population in 2012, included males, incorporated a wider range of life events (including both events that directly involved the study elephant and indirect events that the elephant might have experienced) and assessed both age at first event and the estimated relative risk of elephants’ experiencing that first event. An additional difference is that we did not explore variables associated with rates of survival or mortality, because this study focused only on living animals.

Studbooks estimate the date of wild capture and we can’t know exactly when an imported elephant was separated from its mother. However, the majority of imports (53%, 93/177) involved elephants that were 2 years old or younger, so it is possible that in general they were separated from their mothers at young ages. By contrast, the results of the age at separation analysis demonstrated that the majority (58%) of captive-born individuals were still at the same zoo as their mothers (i.e., had not experienced a transfer or death of the mother) at the time of the study. The average age at separation was 9.3 years for all elephants with a mean age for Africans of 10.1 and for Asians of 7.8, although this difference was not statistically significant. The average ages of separation in our study were similar to those reported by Clubb et al. [46] for captive-born Asian females in European zoos (8.3 years), although in their population, the age at separation tended to be older for captive-born African females (16.3 years). In the wild, females often associate with their natal herd throughout their lives. Thus, separations experienced by captive females could represent potentially stressful life events. Indeed, Clubb et al. [66] found that female Asian calves in zoos that were removed from their mothers at young ages tended to have poorer survivorship. Males in the wild, on the other hand, begin to be excluded from their natal herd or leave voluntarily when they reach puberty, around 8–15 years old [36, 20], an age comparable to the separation age in our zoo population. Furthermore, male elephants in captivity reach sexual maturity at a younger age than those in the wild [20, 68]]. We found that the average ages of separation were not different for males (9.7 years) and females (8.9 years), but the sample sizes were small. However, we noted that the last mother-offspring separation due to transfer for a male occurred in 2011, while the most recent maternal-offspring separation for a female due to transfer occurred in 1998. All mother-offspring separations due to transfer that have occurred since 2000 (n = 7) have been of male offspring. These results suggest that zoo managers are considering female elephant social needs in their approach to population management, keeping mother-female offspring pairs together whenever possible. One study by Evans and Harris [69] found that adolescent males (10–20 years of age) were the most social age group compared to juveniles and adult males in the same study. The researchers assert that this increased sociality is important for the young males to develop necessary social skills. Indeed, it is now understood that bull elephants are not solitary but social animals often seen in large bachelor herds [69]. We need to take this into consideration and provide for their social needs as we would for female only herds.

Over 80% of the elephants in the study experienced at least one inter-zoo transfer in their lifetime. Our analysis revealed demographic patterns in transfer results that were primarily linked to origin. Given that we counted importation as a first transfer event, 100% of imported elephants experienced one or more transfers compared to only 46% of captive-born elephants, and the average age of first transfer was significantly younger for imported (3.3 years) than captive-born (18.8 years) elephants. This indicates a potentially significant difference in the lives of imported elephants insofar as they experience transfers earlier in life and at higher rates than their captive-born counterparts. It is possible that an importation event is qualitatively different from an inter-zoo transfer from the perspective of individual elephant welfare, so future studies should investigate the effects of post-importation transfer events based on origin. However, when the total number of transfers was tested as a risk factor for stereotypic behavior rate performance, it was significant and not confounded by origin [37], which suggests that transfer experience is important to behavioral health for both imported and captive-born elephants [37].

Our results also demonstrated that there was a sex difference with males experiencing higher transfer rates than females, and specifically imported males experienced higher transfer rates than imported females. Given that on average imported males are 4 years younger than imported females, these transfers are occurring more frequently over a shorter time period for males than females. The reasons for this difference are not clear, although a higher transfer rate for imported male elephants in the North American population may be due to the need to move animals for genetic management. For example, there are significantly fewer males than females in the population, and in 2012 only 31 zoos (17 with Asians; 14 with Africans) had bulls on-site [53]. Historically there have been a limited number of specialized facilities equipped to house bulls [62, 70], so when young imported males reached reproductive age they may have been transferred to zoos with more appropriate facilities [68, 71]. However, this situation is changing; the AZA Standards for Elephant Management and Care (approved March 2011) now state that all institutions planning new construction for elephants or modifying existing elephant facilities must include holding space for adult males in their construction/renovation plans. In our study, 43% (29/68) of zoos housed bulls, 38% (11/29) of those housed more than one bull, one of which housed a bull only social group. Additionally, artificial insemination has allowed for increased genetic management of the North American population without the need for transferring elephants between facilities and provided new opportunities to incorporate genetic diversity from wild populations [60, 61]. Unfortunately, many adult bulls are not producing good quality semen, reducing the number of bulls available for breeding programs [61, 72, 73]. Taken together it is unclear how transfers of bull elephants will be affected in the future. But, if the goal is for zoos to maintain a multi-generational social structure, it will necessitate managing larger herds and transferring of males to avoid inbreeding while allowing both females and males opportunities for social learning and development.

### Births and Deaths

Our demographic analyses found that the majority of females in the population were over 30 years of age and had not yet reproduced. Only 25.7% of the African and 32.2% of the Asian females in the zoo population had calved by the end of 2012. Of those, only 51.8% (28/54) had produced more than one calf. A concern for zoo elephants that do not reproduce regularly is that prolonged non-reproductive periods are detrimental to reproductive health [74–76], and in fact in 2012, only 48.4% of African females and 73.3% of Asian females were cycling normally (i.e., not experiencing either acyclicity or irregular cycling) [48]. Low parity is a major challenge for both species, and is hampering efforts to achieve self-sustaining zoo populations [63, 64, 70, 77]. However, the birth rate did not differ between the species in the population, emphasizing that management decisions related to captive breeding play an important role in these findings. For example, in captivity, Asian elephant females begin to cycle at around 5 years of age [48, 53, 78], while African females generally reach puberty around 8 years of age or older [48, 53]. In the North American zoo population, however, the average age at which female elephants first gave birth was 21.3 years for Africans and 16.9 years for Asians.

In a Myanmar study, female elephants involved in logging showed low fecundity up to about 13 years of age, followed by a rapid increase to a peak at age 19 years [79, 80]. In that population, females were fecund into their 50’s, and the oldest gave birth at 65 years of age [79, 80]. In our population, the oldest age at which an Asian elephant gave birth was 40 years of age. Authors of the Myanmar studies found there was no evidence of menopause in elephants, and that females generally remained reproductively active up to death [79, 80]. Comparatively, our data suggests that, of the Asian elephant females reproducing, breeding decisions appear to be maximizing their reproductive potential. In a study of Amboseli African elephants, researchers found that the average age at first reproduction was 13.8 years of age [81]. Furthermore, those that reproduced before the age of 13 had higher age specific fertility rates than those that started reproduction after they were 15 years old, with no differences in survival between these groups [81]. In that population, reproduction did not entirely cease until elephants were over 65 [81], the oldest African elephant to give birth in our population was 36 years old. The average age at first offspring birth would seem to suggest that in general African elephants in zoos begin to breed over 10 years after reaching sexually maturity, and management decisions may be limiting their reproductive potential.

For female elephants in our study population, the average age at first offspring birth was 15 years younger than the average age of being exposed to the first birth of a herd-mate. Although we have no way of knowing if imported females experienced birthing events prior to importation, our data showing that most females were imported at a young age (<2 years of age) suggest that one of their earliest exposures to an infant was probably the birth of their first calf. Overall, the limited exposure to birthing experiences exhibited by imported elephants, in particular imported African females, could result in welfare differences both for themselves and their offspring. Allomothering by younger, nulliparous adolescent females is thought to enhance the stability of family units over time, and the allomothers likely gain experience in rearing young that benefits their own offspring later in life [23, 82]. Our subsequent analyses showed that the presence of calves in herds reduced the risk of stereotypic behavior for both male and female adults [37], and as such addition of juveniles into existing herds through successful breeding may provide an important protective effect from the development of abnormal behavior in the future. Because the goal is to ensure individual animal welfare, health and population sustainability, zoos now recognize the importance of giving females the opportunity to reproduce regularly throughout their lifetime.

Over half of the study females experienced the death of their own offspring. There were additional demographic differences in the pattern of birth and death experiences, with origin once again playing a role. Captive-born female elephants had a higher relative risk of having an offspring born or die, while captive-born males had a higher chance of experiencing a birthing event compared to imported males. In general, captive-born elephants experienced birth and deaths before ever experiencing a transfer. The influence that birth and death events have on individual welfare is unknown, and is likely influenced by factors such as relatedness and length of association. Wild elephants have been observed expressing directed empathetic behaviors when deceased conspecifics or herd mates are encountered [21, 22], which suggests that although a natural process, death can be an emotionally challenging experience for individual elephants. From the perspective of individual elephant welfare, it is unclear how the death of a calf affects the mother or her herd mates, and should be studied further to help us understand the role these biological events play in elephant welfare.

## Conclusions

Our studbook analyses provide unique descriptive data about the zoo elephant population in North America, and also highlight a number of differences in zoo elephant social life events related to species, sex and origin. These differences may help us understand how evolving management strategies can influence specific welfare outcomes. While many of the demographic trends we have highlighted reflect the dynamics of a small, reproductively-managed population that includes individuals from two distinct species, they also indicate there are potentially important differences between the early lives of imported and captive-born elephants that extend beyond species differences and that could have differential long-term effects, for example related to the development of social skills (e.g., via allomothering), the strength of social bonds, or the success of coping strategies later in adult life. Given that there is increasing interest in North American zoos today to promote good welfare for their animals, create self-sustaining populations, and maintain multi-generational elephant herds, variables likely to change are the overall rate of transfers experienced by elephants and the numbers of calves that are captive-born. As the science of elephant management continues to advance, an understanding of the impact of life events will be beneficial to the development of management programs specific to individual elephant needs. As such, future studies will focus on investigating the role of life events on adult elephant temperament, health, and resilience to stress, as described for other species [49, 50, 51].
